# Insect Mating Behaviors: A Review of the Regulatory Role of Neuropeptides

**DOI:** 10.3390/insects16050506

**Published:** 2025-05-08

**Authors:** Alfayo Ombuya, Jianyang Guo, Wanxue Liu

**Affiliations:** 1 State Key Laboratory for Biology of Plant Diseases and Insect Pests, Institute of Plant Protection, Chinese Academy of Agricultural Sciences, Beijing 100193, China; alfayoombuya@yahoo.com; 2 Key Laboratory of Invasive Alien Species Control of Ministry of Agriculture and Rural Affairs, Chinese Academy of Agricultural Sciences, Beijing 100193, China; 3 Kenya Plant Health Inspectorate Service (KEPHIS), Mombasa Regional Office, Mombasa P.O. Box 80126-80100, Kenya

**Keywords:** mating behavior, neuropeptides, courtship, copulation, post-mating behavior

## Abstract

Insect mating behaviors are complex, diverse, and primarily regulated by neuropeptides and their receptors. Neuropeptides are peptide signaling molecules mainly secreted by insects’ central nervous system (CNS) to reach target organs. A substantial body of research on the role of neuropeptides in regulating mating behaviors in insects has been undertaken. This review aims to (1) synthesize existing knowledge on insect mating behaviors, (2) elucidate the neuropeptidergic mechanisms governing these behaviors, and (3) identify knowledge gaps and propose future research directions. The mating process, covering mate attraction, courtship rituals, copulation, and post-mating behaviors, was elucidated with appropriate examples. Additionally, specific neuropeptides involved at each stage of the mating process, their functions, and mechanistic aspects were discussed as demonstrated in research. The review highlights that insects display behavioral dimorphism in the mating process driven by a complex underlying neuropeptidergic mechanism. While previous publications have generally addressed the role of neuropeptides in insect behavior, none has intensively and methodically examined their role in mating behaviors. In this review, we synthesized 18 neuropeptides that we found to regulate mating behaviors in insects. We note that some of the neuropeptides are malfunctional in their regulatory roles, while others are specific. We also note that these neuropeptides execute their regulatory functions through the G protein-coupled receptor (GPCR) signaling pathway but may take different routes and messengers downstream to effect behavioral change. Neuropeptides also interact with other regulatory systems, such as the endocrine system, to discharge their functions. Given their significance in mediating mating and reproduction, targeted manipulation of the signaling system of neuropeptides could serve as viable targets in the production of ecologically friendly pest management tools. Tools that could disrupt the mating process would be applied in crop production systems to reduce the population pressure of destructive pests, consequently reducing the urge to use chemical pesticides that are ecologically unfriendly. Our findings not only advance the understanding of neuropeptide-mediated mating regulation but also highlight their potential as eco-friendly pest control targets.

## Simple Summary

Mating is a fundamental behavior of animals in perpetuating species. The mating behaviors of insects are complex and primarily regulated by small molecules called neuropeptides. Here, we highlight what is already known about insect mating behaviors and the role played by neuropeptides. In our findings, we note that the mating process of insects entails mate attraction, courtship, copulation, and post-mating behaviors. The 18 neuropeptides discussed in this review are known to regulate mating behaviors and greatly vary among insects. The Neuropeptides vary in mechanistic aspects, and some work closely with other physiological and neurocircuit systems. Moreover, many details about neuropeptides remain unknown, especially in many economically important insects. Given their significance in successful mating and reproduction, neuropeptides remain potential targets for developing safe and sustainable pest control products.

## 1. Introduction

**Figure 1:**
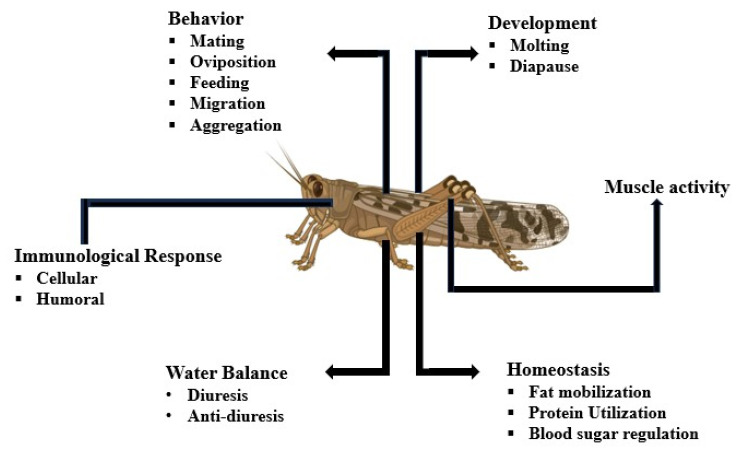
Nearly every physiological function in insects, including immunological responses, pheromone production, diuresis, ecdysis, and muscle activity regulation, is mediated by insect neuropeptides [1,4], Created in BioRender. guo, j. (2025) https://BioRender.com/zeb0u85 (accessed on 28 April 2025).

Insect mating behavior is a cornerstone of species survival and evolutionary success. Deciphering its regulatory mechanisms not only deepens our understanding of insect biology but also opens avenues for neurobiological innovation and sustainable pest control strategies. Insect mating involves several behavioral and physiological regulations that are evolutionarily pressured to promote successful reproduction. The wide range of mating behaviors is known to vary significantly across various species. Neuropeptides—small peptide molecules secreted by neurosecretory cells—regulate insect physiology through G protein-coupled receptors (GPCRs) [1]. These tiny peptide molecules are crucial for growth, nutrition, reproduction, metabolism, and many other processes (Figure 1) [1,2]. Neuropeptides regulate various aspects of insect life by functioning as neurotransmitters, neuromodulators, hormones, or growth factors [1]. They coordinate intricate behaviors responding to internal and external signals by interacting with specific receptors in the central nervous system and other peripheral tissues. In insect reproduction, neuropeptides are crucial modulators of mating behaviors [3].

In addition to expanding our understanding of insect biology, the understanding of how neuropeptides regulate mating behavior in insects is pivotal in the creation of novel tools and approaches in neurobiology and pest control. Researchers have examined the role of neuropeptides in the mating behaviors of various insect species. Their findings have revealed both conserved and species-specific mechanisms. While previous publications have addressed the role of neuropeptides in insect behavior, none has intensively and methodically examined their role in mating behaviors. In this review, we aim to provide an overview of how insects engage in mating behavior and how neuropeptides regulate them. We begin by highlighting the mating process, ranging from mate attraction, courtship rituals, and copulation to post-mating behaviors, with appropriate examples. Next, we review specific neuropeptides involved at each stage of the mating process with relevant examples as demonstrated in research, and we conclude by discussing applications in pest control. Lastly, we point out knowledge gaps with suggestions on the new directions for neuropeptide research.

## 2. How Insects Are Adapted to Mating Behavior

**Figure 2:**
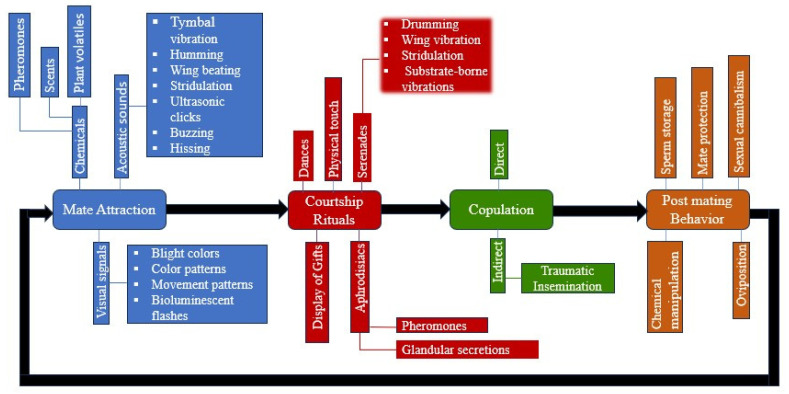
Insects exhibit a complex continuum of mating behaviors, encompassing mate attraction, courtship rituals, copulation, and post-mating activities. These behaviors are evolutionarily ingrained, uniquely adapted for survival, and vary across species. While some insects use chemical cues to attract mates, others utilize enticing songs. Some engage in intricate dances, while others present gifts to "sweep her off her feet”. Created in BioRender. guo, j. (2025) https://BioRender.com/0lismvv (accessed on 28 April 2025).

Insect mating encompasses mate attraction, courtship rituals, copulation, and post-mating behaviors (Figure 2).

### 2.1. Attraction of Mates

Insects utilize multimodal sensory cues, including chemical (pheromones), acoustic, and visual signals, to locate and attract mates.

#### 2.1.1. Chemical Communication (Pheromones)

Insects utilize a variety of chemical signals to attract mates, with pheromones being particularly important for long-distance communication [5]. Female moths and butterflies release sex pheromones capable of attracting males from several kilometers away. The pheromones are released from specialized glands in the females, and males can detect them using highly sensitive antennae. Female silkworm moths (*Bombyx mori*) are known to release bombykol pheromone, a volatile 16-carbon alcohol to attract male mates [6], while female gypsy moths (*Lymantria dispar* (L.)) release a sex pheromone identified as (Z)-7,8-epoxy-2-methyloctadecane (synthetic; disparlure) [7]. Male monarch butterflies (*Danaus plexippus*) employ a unique strategy to attract females by producing specific scents. The specific scents are released by specialized structures called hair-pencils located at the tip of the abdomen, and they are used to communicate the readiness and desirability of a mate [8]. The Colorado potato beetle (*Leptinotarsa decemlineata*) exhibits an intriguing chemical communication strategy by releasing both aggregation and sex pheromones. The male Colorado potato beetle produces an aggregation pheromone (S)-3,7-dimethyl-2-oxo-oct-6-ene-1,3-diol (also referred to as (S)-CPB I), which attracts both males and females to specific locations for feeding and mating activities [9]. Male Fruit flies emit pheromones to entice virgin females and other males to mate [10]. To attract females, the male fruit flies (*Drosophila melanogaster*) use cuticular hydrocarbons (CHCs) as a crucial part of mating behaviors, i.e., mate selection and female receptivity [11]. Queen honeybees (*Apis mellifera*) produce the queen mandibular pheromone (QMP), which attracts drones during the queen’s mating flights [12]. German cockroach females (*Blattella germanica*) use volatile and contact sex pheromones to attract males. Pygidial glands of the females release the volatile pheromone called blattellaquinone, which can draw males in from a far distance [13,14]. Volatile pheromones are also employed by female Tsetse flies (*Glossina* spp.) to attract and select mates [15]. Methyl palmitoleate (MPO), methyl oleate, and methyl palmitate compounds are among the chemicals identified as responsible for promoting rapid mating behavior in male tsetse flies [15].

Plant volatiles play a significant role in the process of mate-finding in numerous insect species, including herbivores, pollinators, and parasitoids [16]. The release of these plant-derived volatiles from leaves, flowers, and fruits serves as rendezvous sites for mate-seeking insects and may elicit the release of pheromones. According to Xu and Turlings [16], certain insects can locate mates quickly and effectively by combining pheromones and plant volatiles. The aggregation pheromones released by female bark beetles (*Dendroctonus ponderosae*) draw males and females to appropriate host trees for feeding and mating. The trans-verbenol released by females (*D. ponderosae*) is very attractive to both sexes when combined with volatiles from the host tree [17,18].

#### 2.1.2. Acoustic Signals (Sounds)

Some insect families and orders use acoustic signals to attract mates from long distances. Different mechanisms, including tymbal vibration, wing beating, and stridulation, are used to produce the sounds. Orthopterans, i.e., grasshoppers, crickets, and katydids, make sounds to attract mates. Male crickets (*Gryllus* spp.) and katydids (*Tettigonia viridissima*) stridulate or rub their forewings together to make seductive chirping species-specific songs to attract females over long distances [19]. Male grasshoppers also produce sounds by rubbing their hind legs against their wings to attract mates. Cicadas (Hemiptera: Cicadidae) use tymbals to produce loud mating call sounds [20]. Periodical cicadas (*Magicicada* spp.) and annual cicadas (*Tibicen* spp.) produce some of the loudest insect sounds. These sounds can be heard over long distances, which can be hundreds of meters [21]. Certain Coleopterans of the families Scarabaeidae (scarab beetles) and Cerambycidae (longhorn beetles) make sounds by tapping on objects or rubbing body parts together (stridulation) to attract mates. Both male and female longhorn beetles (Cerambycidae) produce sounds through stridulatory organs, which include a scraper on the pronotum and a file on the mesoscutum [22]. The humming sound produced by the wing vibrations of the female mosquitoes helps males locate the females for mating [23]. *D. melanogaster* males use wing vibrations to produce unique acoustic signals to attract females, with recent studies revealing up to three different patterns of acoustic songs [24]. The male tiger moths (*Bertholdia trigona*) utilize ultrasonic clicks to attract females and jam bat signals to avoid predation [25]. When digging from their underground nests to the soil surface, females of digger wasps (*Bembix rostrata*) and solitary bees (*Colletes cunicularius*) emit volatile odors and buzzing sounds that serve as cues for patrolling males to estimate the approximate location of the digging virgin females [26]. To attract females, male Madagascar hissing cockroaches (*Gromphadorhina portentosa*) produce hissing sounds [27]. Moreover, features of the hisses vary on the dominant frequency, duration, and rate [27].

#### 2.1.3. Visual Displays

Visual displays play a significant role in the mating behavior of certain insects. Bright colors, patterns, or particular movements are displayed to entice mates. In signaling males about their readiness to mate, the female firefly flashes its bioluminescent backside light to attract a suitable mate who responds by specific flash patterns [28]. Dragonflies and damselflies perform aerial acrobatics to attract mates. In calling and enticing females, male dragonflies (*Sympetrum* spp.) engage in flight displays [29]. The mating behavior of the rhinoceros beetle (*Xylotrupes gideon*) is characterized by aggressive male competition intertwined with specific displays to attract receptive females. To enhance their chances of mating, males show their power and desirability by using their horns to force competitors off branches or out of their territory [30]. Upon overcoming rivals, a successful male uses its horns to perform visual displays that may attract the female into mating with him [30]. Virgin mantis females, *Hierodula patellifera* (Dictyoptera: Mantidae), exhibit a characteristic calling posture [31]. To attract males, a female flexes the abdomen away from the wings and exposes the dorsal surface by curling it ventrally when holding the body beneath a branch or leaf with pumping movements accompanying the curling [31].

### 2.2. Courtship Rituals

Insect courtship entails elaborate behaviors designed to attract females, and these behaviors are species-specific. Courtship behaviors are numerous, entailing serenades, dances, physical touch, displays of gifts, and aphrodisiacs depending on the species [23].

#### 2.2.1. Serenades

Serenades are vibrational songs or acoustic signals that insects produce to entice potential mates. Insects also use serenades to evaluate the quality of their mates and to coordinate mating habits [32]. Drumming, wing vibrations, substrate-borne vibrations, and stridulation are the forms of serenades [33,34,35]. Courtship behaviors of the silkworm moth (*B. mori*) are a complex interplay of olfactory cues and specific motor actions. Upon recognizing sex pheromones from a receptive female, the male silkworm moth (*B. mori*) flies towards the female and performs a series of courtship displays. Firstly, the male will produce a wing song by flapping their wings to enhance the auditory aspect of the courtship [36]. The male will then use its forelegs to touch or grasp the female’s abdomen in attempted copulation, signaling an intention to mate [36]. Males of the house cricket (*A. domesticus*) call females from a distance through chirping (stridulation), which is performed by rubbing wings together. Each male has a distinctive way of advertising his presence and mating fitness by singing a unique courtship song [37]. At times, males may engage in singing rivalry where they want to outdo each other in singing to attract females. According to studies, females frequently base their partner selection on the wooing song’s qualities, such as its length and output [37,38]. The courtship behavior of cicadas combines sound production, visual displays, and tactile interactions that vary in complexity and style [39]. Male cicadas use tymbals, which are unique drum-like structures on their abdomens, to make loud calls to attract females. The pattern of these calls and their intensity can draw in females from a distance and reveal a male’s level of fitness [40]. Each species of cicada has a unique song to prevent cross-species mating. Some species, such as the periodical cicadas (*Magicicada* spp.), can have thousands of males calling simultaneously in large choruses varying in duration and frequency [39]. Female cicadas do not sing but respond to calling males by using soft wing flicks and distinguishing calls from different males, normally preferring those with complex patterns and higher frequency [39]. Upon hearing the soft wing flicks, males adjust their courtship calls accordingly while moving closer to the receptive female. Males of some species of cicadas also engage in body movements and visual signals. Larger males with stronger calls are generally more attractive to females.

#### 2.2.2. Dances

Courtship dances in insects feature complex motions, patterns, or displays intended to woo mates, show off physical prowess, or create pair bonds. The courtship behavior of the dragonfly (*Pachydiplax longipennis*) is a combination of aggressive pursuit and stunning aerial displays [41]. Courtship occurs both in-flight and while perched. Males actively search for receptive females and engage in aerial chase [41]. Chasing involves impressive flight displays that demonstrate agility and fitness. Upon successful reception by a female, the male will grasp the female to copulate [42]. The honeybee (*A. mellifera*) engages in courtship during nuptial flights. Queens mate during specific periods of their lives shortly after emerging as adults. To mate, drones gather in designated areas known as drone congregation areas (DCAs), where they perform aerial dances to attract virgin-receptive queens [43]. When a queen enters a DCA, she is competitively pursued by drones and impressed through flight dances. A successful drone mates with the queen but dies in the mating process due to the rupture of his genitalia, leaving his endophallus bearing the sperm inside the queen [43].

#### 2.2.3. Physical Touch

Insects employ physical touch to assess potential mates, synchronize mating moods, and stimulate reproductive readiness [44]. This tactile communication can involve the antennae, legs, wings, or specialized body structures. The courtship behavior of the Japanese beetle (*P. japonica*) is defined by a mix of mating tactics, male competition, and specific rituals. The beetle displays promiscuous mating, where both males and females can mate with multiple partners in a phenomenon commonly referred to as “scramble competition” [45]. To gain access to females, males actively compete with one another through behavioral and physical contests, where larger males are more likely to succeed in taking receptive females [45]. Successful males engage in particular courtship activities, including tactile behavior, mounted courtship, and attentiveness to the female, to win mating acceptance [45,46]. The male German cockroach (*B. germanica*) combines the use of pheromones and tactile behaviors to court females. A male initiates courtship by following a female and coming up behind her, occasionally tapping her with their forelegs or antennae to stimulate her response [47]. Receptive females release contact sex pheromones that influence male behavior during courtship [48]. Males may also exhibit particular postures and motions that indicate their interest and preparedness for mating [49].

#### 2.2.4. Display of Gifts

Certain male insects offer nuptial gifts to females during courtship or mating. Nuptial gifts can be in the form of food, body parts, or secretions from glands intended to attract females and maximize sperm transfer [50]. Increasing female receptivity, extending copulation, or improving reproductive output are some of the ways nuptial gifts enhance mating success. During mating, the male of some cricket and bush-cricket (Orthoptera; Ensifera) species gives the female an elaborate spermatophore containing spermatophylax (a nutrient-rich edible portion) to keep the female occupied while sperm transfer occurs [51]. Dance flies (Diptera: Empididae) exhibit complex mating behavior that involves offering nuptial gifts. This species’ males court females by presenting them with prey items, which can influence the choice of mate and the success of copulation [52]. The likelihood of mating is frequently increased by the size of the gift [53].

#### 2.2.5. Aphrodisiacs

To increase receptivity in a potential mate, certain male insects produce chemical compounds or other substances referred to as aphrodisiacs. Aphrodisiacs can be in the form of pheromones or glandular secretions. Studies have revealed that during courtship, male butterflies of different species release aphrodisiac compounds. The male of the queen butterfly (*Danaus gilippus*) produces an aphrodisiac secretion from the abdominal hair-pencils, which they apply in dusting potential mates via the female’s antennae [34]. If the dusted female becomes receptive, the male again releases the compound to reinforce her readiness. Once she is fully prepared, they fly together to a suitable location to mate. Male *D. melanogaster* uses a mix of vibrational cues, sex pheromones, and visual signals to court females [54]. Males vibrate their wings to produce a courtship song, and they frequently tap the female’s body with their forelegs to express their interest [54]. Moreover, the males release a pheromone to signal the females their readiness to mate [11].

### 2.3. Copulation

Copulation is the actual mating process that involves the transfer of sperm from the male to the female. The majority of species practice direct copulation, while in other species, copulation occurs through indirect means. Direct sperm transfer is the most common among species. In direct copulation, male insects transfer sperm into the female’s reproductive tract through the aedeagus (equivalent to a penis) [55]. The sperm may be transferred directly to the spermatheca for storage or indirectly through a spermatophore [55]. During copulation, male beetles insert their aedeagus directly into the female’s bursa copulatrix to transfer sperm cells [56]. In butterflies, copulation involves sperm transfer via a spermatophore from the male into the female genital tract. Some insects practice indirect copulation, which occurs without physical contact between sexes. The males of silverfish and springtail place a spermatophore on a surface, and the female later picks it up via her genital opening [57]. In bedbugs (*Cimex* spp.), copulation occurs via a unique technique known as traumatic insemination, in which the male injects sperm directly into the female’s body cavity by piercing her abdomen with his aedeagus [58]. Traumatic insemination bypasses the conventional process of transferring sperm cells through the genital opening, and bedbugs’ mating behavior lacks typical courtship rituals. Depending on the insect species, copulation can last anywhere from a few seconds to several hours. Several variables, including mate-guarding behavior, sperm competition, and nutrient transfer, may be the causes that affect the duration of copulation.

### 2.4. Post-Mating Behaviors

To maximize reproduction success, insects display a variety of post-mating activities after copulation. Sperm storage, mate protection, chemical manipulation, oviposition, and occasionally sexual cannibalism are examples of post-mating activities undertaken by various species of insects.

Some female insects store sperm in the spermatheca and use it over time to fertilize eggs. Honeybee (*A. mellifera*) queens can store and maintain the viability of sperm for many years, enabling them to fertilize thousands of eggs without needing to mate again [59]. Sperm selection and utilization processes in butterflies and moths enable females to control which male’s sperm fertilizes their eggs [60]. In certain species, males stay with the female after mating to prevent other males from mating with her. The male may guard the female physically or may engage in prolonged copulatory contact with her. Male dragonflies and damselflies remain attached to the female while she lays eggs to guard her from mating with other males [61]. Certain insect species create mating plugs inside the female reproductive tract to block reproduction with competitors. For example, in *D. melanogaster*, the male seminal fluid creates a mating plug inside the female bursa, preventing sperm entry or the female from remating [62]. Some insects employ sperm competition tactics to reduce reproduction success with rival males. Male damselflies (*Hemiphlebia mirabilis*), for example, use specialized structures to remove a rival’s sperm from the female genitalia before depositing their own [63]. Certain male insects transfer chemical substances that affect female behavior, making her less interested in mating. A study by Denis and Claisse [64] revealed that male *D. melanogaster* produce proteins in their semen to suppress female receptivity and remating. Studies have also shown that due to some chemical compounds passed by the male, mated female butterflies and moths of polyandrous species frequently show a period of non-receptivity [65]. In certain species, the male is consumed by the female either during or after mating to supply nutrients for the generation of eggs. Female praying mantises (*Hierodula membranacea*) and spiders have been observed to consume their male companions during or after mating [66,67]. The primary concern of insects after mating is to place eggs in an area where they are not vulnerable to predators. Egg-laying techniques and patterns vary greatly and are unique among species, but typically, females lay their eggs in, on, or next to the juvenile stage’s diet. For example, mosquitoes lay eggs in standing waters to enhance larval development time [68].

## 3. Role of Neuropeptides in the Mating Process

Insect behaviors, whether basic or complex, are regulated by neuropeptides [4].

### 3.1. Role in Choice of Mates, Attraction, and Courtship

Neuropeptides have been shown to affect receptivity, courtship displays, and mate appeal in insects. The various neuropeptides mediating these processes are discussed.

#### 3.1.1. Effect on Receptivity

##### Pheromone Biosynthesis-Activating Neuropeptide (PBAN)

Pheromone biosynthesis-activating neuropeptide (PBAN) is crucial in mating behavior and is primarily found in lepidopteran insects (like moths). PBAN is specific to females and regulates the release of sex pheromones to attract mates [69,70]. It promotes calling behavior and receptive postures in female moths and ensures species-specific pheromone blends to avoid cross-species mating, thus promoting mating success [71]. PBAN is a 33-amino acid neuropeptide member of the PBAN/pyrokinin peptide family, which is distinguished by the conserved C-terminal motif of FXPRLamide, which is essential for its role [72]. PBAN is produced in the subesophageal ganglion (SEG) of the insect brain and released into the hemolymph through the neurosecretory cells [73]. PBAN neurons are found in the SEG and project to either the corpora allata or the corpora cardiaca, which are neurohemal organs. The signal transduction of sex pheromone synthesis is by PBAN acting on the pheromone gland epidermal cells located at the posterior abdomen of the female insect and binding to a G-protein coupled receptor (PBAN-R) on these cells [71]. The binding of PBAN to PBAN-R activates signaling cascades via an influx of extracellular Ca^2+^, which stimulates enzymatic pathways that lead to sex pheromone biosynthesis [74]. PBAN neurons in the SEG are influenced by circadian signals. The release of PBAN is synchronized with the circadian clock, thus aligning sex pheromone production with the time of day when mating is likely to happen, usually during scotophase [72]. The release of PBAN production significantly decreases after mating, which contributes to the loss of female receptivity [72]. Some studies have also shown that PBAN production is tied to the nutritional and hormonal states of the insect. For instance, the juvenile hormone (JH) upregulates PBAN expression [72,75]. Studies have also shown that PBAN sequences and functions in lepidopteran species are highly conserved despite divergence in pheromone chemical structures, suggesting strong evolutionary pressure to retain their function in mating behavior [76]. Male attraction is curtailed, and pheromone production is reduced or eliminated when PBAN or its receptor is knocked down. A study on *P. xylostella* found that RNAi of the PBAN receptor (Plx-PBANr) significantly reduced the biosynthesis of sex pheromones, resulting in a decrease in male attraction and a cessation of mating behavior [77]. Additionally, when the PBAN gene was edited via CRISPR/Cas9-mediated editing, females were found to be less attractive to males, which resulted in the disruption of mating activities. Similarly, in another study on the fall armyworm (*S. frugiperda*), females were found to be less attractive to males when the PBAN gene was altered using CRISPR/Cas9-mediated editing, which also disrupted mating activities [78].

##### Tachykinin-Related Peptides (TRPs)

Tachykinin-related peptides (TRPs) are a family of neuropeptides found in invertebrates. Functionally and structurally, TRPs are analogous to the vertebrate tachykinins. TRPs are produced in the CNS (brain regions, thoracic ganglia, and abdominal ganglia) and peripheral tissues and are characterized by a conserved motif, C-terminal pentapeptide FX_1_GX_2_Ramide [79,80]. In locusts (*Locusta migratoria*) and cockroaches (*Leucophaea maderae*), midgut endocrine cells additionally secrete TRPs as circulating hormones [81,82]. TRPs are multifunctional, modulating locomotion, aggression, feeding, circadian cycles, and, most importantly, sexual behavior. In crickets, TRPs initiate mating calls and modulate sex pheromone release [83]. Studies have demonstrated the role of TRPs in suppressing insect courtship. In fruit flies (*D. melanogaster*) females, TRPs released from a cluster of eight to ten cells located in the *c929*-labeled SEZ region within the subesophageal zone regulate the detection of gustatory pheromone required in the suppression of pheromone-triggered courtship [84]. TRPs also induce the release of adipokinetic hormone (AKH) from the corpora cardiaca in locusts [82]. AKH is critical in mobilizing lipids, carbohydrates, and proline from stores during energy-demanding activities like mating [85]. TRPs specifically interact with a subfamily of structurally related G protein-coupled receptors to produce biological effects [80]. In *D. melanogaster*, the signaling pathway involves TRPs binding to Tachykinin Receptor 86C (TkR86C). TkR86C activation triggers G-protein-coupled signaling, which in turn activates phospholipase C (PLC), raises intracellular Ca^2+^ and second messengers like Inositol trisphosphate (IP_3_), and modulates ion channels and neurotransmitter release. The changes trigger neuronal excitability downstream, influencing behavioral decisions. TRPs are widely conserved in insects, including cockroaches, ants, locusts, flies, beetles, and moths. The FX_1_GX_2_Ramide signature is conserved in all the insect TRPs [79]. Consequently, reproductive strategies could differ, but the TRP neuromodulatory system in many insects remains functionally conserved.

##### Natalisin (NTL)

Natalisin (NTL) is unique to insects and crustaceans and is a member of the tachykinin-like peptide superfamily that modulates insect reproduction and sexual behavior [49]. It is named NTL because of its significant role in natal functions, especially mating and reproduction. Like TRPs, NTL also has a conserved C-terminal motif FxGxRamide but differs in certain specific sequence features. NTL is highly expressed in mature adults because of its role in mating and is produced within the central nervous system, particularly in the brain, ventral nerve cord, and the SEG [49,86]. The expressing neurons of NTL are located in the neuromodulatory centers of the insect brain, pars intercerebralis, SEZ, and abdominal ganglia. NTL has been shown to modulate receptivity. In a study, when NTL was silenced in both males and females of *S. frugiperda* via RNAi, a significant reduction in sexual activities was observed, attributable to the reduced calling rate of females and the declined courtship rate of males [49]. Besides promoting receptivity, NTL modulates fecundity. In a study, fecundity was significantly reduced upon NTL knockdown on *T. castaneum* [87]. NTL’s transduction signal is via a specific GPCR-natalisin receptor (NTLR) [87]. The binding of NTL to NTLR triggers G-protein-coupled signaling, which triggers changes in Ca^2+^ mobilization, cAMP levels, and MAPK (Mitogen-Activated Protein Kinase) pathway activation. The changes in these pathways influence neuronal excitability and hormonal regulation, resulting in changes in behavior. NTL and its function of promoting mating behavior are highly conserved in insects and crustaceans, implying that it could be an arthropod-only survival innovation. The fact that RNAi silencing of NTL or its receptor consistently results in impaired mating behavior across several insect orders suggests that there is a strong evolutionary selection to keep its function.

#### 3.1.2. Courtship Rituals

##### Neuropeptide F (NPF)

Neuropeptide F (NPF) is a key neuropeptide in insects that plays a significant role in sexual behavior and reproduction. NPF is an insect homolog of vertebrate neuropeptide Y, produced in the CNS, particularly the brain and the ventral nerve cord [3,88]. The pars intercerebralis, lateral protocerebrum, and mushroom body output neurons (MBONs) are a particular subset of neurons in the brain and ventral nerve cord that express NPF. NPF is short, conserved, and characterized by a C-terminal end with an RPRFamide motif and regulates a variety of neuromodulatory functions, including feeding, stress management, and sexual behavior [89]. NPF affects courtship intensity and sexual drive in *D. melanogaster*, especially in males [90]. Even when deterrents are present, males of *D. melanogaster* with higher NPF levels exhibit improved courtship and a greater desire to mate. In a study on desert locusts (*Schistocerca gregaria*), NPF was also observed to promote courtship, among other physiological behaviors [91]. When given daily injections of trNPF, the adult males showed courtship behavior earlier, whereas RNAi knockdown of the Schgr-NPF precursor transcript prevented or postponed the courtship behavior [91]. NPF and PDF neurons work together to promote prolonged mating duration in male *D. melanogaster* [92]. In a study, the males were also observed to extend their mating period when other males were present, a phenomenon called rival-induced longer mating duration (LMD). The signaling of NPF and PDF with their receptors was critical for LMD to occur [92]. The mechanistic aspects of NPF entail particular biochemical interactions through its G-protein-coupled receptor, NPFR1 [89]. When NPF binds to NPFR1, adenylate cyclase is inhibited, lowering cyclic adenosine monophosphate (cAMP) levels, which in turn affect downstream effectors, causing neuronal excitability and behavioral expression. This NPF-NPFR signaling axis is lauded as one of the most functionally and structurally conserved neuropeptide systems in invertebrates [93].

##### Diuretic Hormone 31 (DH 31)

Similar in structure and function to vertebrate calcitonin gene-related peptide (CGRP), Diuretic Hormone 31 (DH31) is a 31-amino acid peptide with a highly conserved C-terminal amide group. DH31 traditionally regulates water balance, but recent studies show that DH31 mediates mating and reproduction behaviors [94]. In a study, DH31 was identified as key in promoting the transition of male *D. melanogaster* from feeding to courtship [95]. In males that were starving, it was observed that feeding took precedence over courtship. However, the order quickly reversed within a few minutes when the males consumed protein-rich foods. DH31 was found to be responsible for the molecular mechanism behind the transition from feeding to courtship. Besides, DH31 also plays a key role in triggering reproductive dormancy during unfavorable conditions, and during this period, egg development is halted. In a study on the female *D. melanogaster*, low temperatures and short days caused reproductive dormancy by reducing the production of juvenile hormone (JH) in the corpus allatum (CA), an action mediated by DH31 [96]. DH31 is expressed in the small and large lateral ventral neurons of the circadian clock circuit, the ventral nerve cord, the enteric ganglia, and the male accessory gland-innervating neurons. The molecular mechanism of DH31 involves binding to its receptor DH31-R, a G-protein-coupled receptor [94]. DH31-R activation increases intracellular cAMP, and the downstream effects of the cAMP-PKA cascade lead to neuronal excitability in clock neurons, visceral muscles, and the sensory processing centers, resulting in behavioral expression. DH31 has been seen to be conserved across insects, with its homolog peptides characterized in nematodes and vertebrates. Functionally, DH31 is evolutionarily deeply conserved to modulate reward signaling, circadian timing of mating, and sexual satiety to inhibit remating [94].

##### Proctolin (Proc)

Proctolin (Proc) was first characterized in the American cockroach (*Periplaneta americana*) in the 1970s and is a pentapeptide with the Arg-Tyr-Leu-Pro-Thr sequence [97,98]. It is unique to the arthropods, though together with its receptor, it is absent in the genomes of Hymenopterans, Lepidopterans, and the yellow fever mosquito (*Aedes aegypti*) [97,99]. The primary role of Proc in arthropods is to control muscle movements, enhance gut motility and sexual behavior, and act both as a neurotransmitter, neuromodulator, and hormone [97,100,101,102]. In locusts, Proc also serves as a releasing factor of juvenile hormone (JH) and adipokinetic hormone (AKH) [99], which are critical in the mating process. In addition, studies also suggest that Proc modulates fecundity. The ovipositor muscles of the female tobacco hornworm (*Manduca sexta*) were shown to contain Proc, suggesting its role in promoting egg-laying [103]. Similarly, a study on locusts demonstrated the role of Proc in improving muscle contractions that are pivotal in sustaining the oviposition digging rhythm in locusts [104]. Copulation also requires meticulous coordination of muscle movements enabled through proctolin [97]. Proc is produced in the central nervous system, including the brain and ventral ganglia, and is widely expressed in the visceral and skeletal muscles, including the reproductive tissues [105,106]. To execute its function, Proc binds to its receptor ProcR, which is a GPCR. ProcR activates the phospholipase C pathway and protein kinase C, which increases IP_3_, diacylglycerol (DAG), and intracellular Ca^2+^, leading to increased muscle contraction, neuron excitability, and behavior expression [98,107]. In a study, the injection of phorbol ester into the Proc-sensitive neural sites of the male grasshopper (*Chorthippus biguttulus)* triggered courtship singing [98]. The finding suggests its role in insect courtship in addition to the modulation of mating. In the male kissing bug (*Rhodnius prolixus*), Proc causes muscle contractions of reproductive structures, which facilitate the transfer of seminal fluid and sperm ejaculation during copulation [108]. Proc occurs across many insect orders, with structurally similar peptides occurring in some members of crustaceans (lobsters and crabs) and myriapods (centipedes). However, Proc appears to be an evolutionary innovation of the invertebrates performing analogous functions like oxytocin in the vertebrates.

##### SIFamide (SIFa)

SIFamide (SIFa) is a highly conserved neuropeptide in arthropods, secreted by the CNS specifically in four highly specialized neurons, pars intercerebralis (PI) of the brain, with its axons projecting into the mushroom body, lateral protocerebrum, and the central complex [109,110]. It comprises four conserved amino acids at its C-terminal end, thus its name SIFamide (Ser-Ile-Phe-NH₂). It is precise and powerful in modulating sexual behavior, feeding, sleep, and circadian rhythms in insects [109,111,112]. The signaling pathway of SIFa involves binding with its receptors (SIFR), which is a GPCR. SIFR activation initiates the Gq signaling pathway, which stimulates the increase in cAMP levels, IP_3_, DAG, and intracellular Ca^2+^, leading to neuronal excitability, synaptic plasticity, and behavior expression [110]. SIFa affects pheromone detection and sexual attraction in fruit flies (*D. melanogaster*), which in turn affects male courtship behavior [113]. When SIFa is knocked down, males that lack SIFa or its receptor exhibit bisexual tendencies, implying that SIFa is essential for distinguishing appropriate mates [113]. In addition, males also exhibit male-to-male courtship behavior if neurons carrying a SIFaR-gal4 transgene express SIFamide receptor RNAi or are destroyed by the apoptotic protein reaper [113]. SIFa is also essential for integrating several internal and external cues to coordinate multiple tasks, including mating duration [114,115]. For instance, in *D. melanogaster*, SIFa neurons and sNPF neurons work together in a feedback loop to modify behaviors like shorter mating duration (SMD) and longer mating duration (LMD) [114,115]. Mating duration is shortened in males when SIFa neurons are activated [115]. SIFa is extremely conserved among many species of arthropods, and the evolutionary strategy to deploy a small number of neurons to modulate broad behavioral outcomes points to the crucial role of the neuropeptide [109].

##### Orcokinin (OK)

First identified in the crustacean *Orconectes limosus* (thus the name), OKs are a family of myotropic neuropeptides that have more recently been found in insects, arachnids, and annelids [116]. OK genes produce two distinct mature peptides, orcokinin-A (OK-A) and orcokinin-B (OK-B); however, OK-A is generally longer than OK-B. OK are characterized by a conserved N-terminal Asn-Phe-Asp (NFD) sequence. They are produced in the CNS and the midgut and modulate a variety of physiological processes, including ecdysteroidogenesis, locomotion, and reproduction [116,117]. OK is expressed in the abdominal ganglia, brain-optic lobe interface, neurosecretory cells, and the enteric nervous system. The signaling mechanism of OK is not well understood but is presumed to be through GPCRs coupled with Ca^2+^ signaling pathways. OK is confirmed as essential in the courtship behavior of insects. In a study, male-to-male courtship, which is even uncommon in the wild *D. melanogaster*, was shown to be rampant when the OK gene was knocked down via RNAi [117]. Besides its role in courtship, OK promotes fecundity by regulating vitellogenin transcription, as observed in the cockroach (*B. germanica*) [118]. Similarly, silencing the OK gene in female *D. melanogaster* decreases egg production [117]. The evolutionary conservation of OK across various invertebrates points to its significance in mediating physiological functions.

##### Leucokinin (LK)

Leucokinins (LKs) are tiny neuropeptides initially characterized in cockroaches (*Leucophaea maderae*) [119]. LKs are found in many insect orders and are distinguished by the highly conserved C-terminal pentapeptide motif, FXSWGamide. LKs are produced in the lateral horn (projecting to the mushroom body and protocerebrum), subesophageal zone (SEZ), and the ventral nerve cord (CNC) and expressed in the Malpighian tubules. Its signaling mechanism entails LK binding to Leucokinin Receptor (LKR), a GPCR, which initiates the Gq signaling pathway, which stimulates the increase in IP_3_ and intracellular Ca^2+^, leading to neuronal excitability and behavior depending on the state [120]. In dehydration or starvation, LK inhibits mating to conserve energy, while in hydrated and fed states, LK enhances arousal to encourage mating behavior. They are multifunctional, modulating various physiological processes and behaviors in insects and other invertebrates. LK neurons in *D. melanogaster* regulate water homeostasis, feeding, sleep, metabolism, and memory formation [119,121]. LK is an essential modulator of the balance between a courtship touch and defense against body contact [122,123]. When a male courted virgin females in a study on *D. melanogaster*, the females’ defensive response to stimuli was compromised; however, after mating, the response improved. The study revealed that the switch between defensive reaction and receptivity to a courtship touch is mediated by the activation of uterine neurons, which consequently activate an LK-dependent pathway [122]. Across insect orders, LK is conserved, and the C-terminal motif (FXSWGamide) is nearly constant, suggesting a strong evolutionary selection due to its vital function in modulating homeostasis and behavior.

#### 3.1.3. Mate Appeal

##### Pigment-Dispersing Factor (PDF)

The pigment dispersing factor (PDF) is a highly conserved 18-amino-acid, α-amidated neuropeptide [124]. PDF is essential for the modulation of numerous physiological processes, including circadian rhythms, locomotor activity, courtship behavior, and the production of male sex pheromones [92,125,126]. PDF’s role in modulating circadian timekeeping is well studied and understood. However, it is shown that PDF affects sex pheromone biosynthesis and mating behavior, aligning it with the circadian clock. In a study by Krupp and Billeter [127] on *D. melanogaster*, it was revealed that disruption of the PDF signaling pathway reduced male sex pheromone production, consequently affecting mate appeal. For insects that mate at certain peak times, for instance, at dawn or dusk, PDF sets this behavioral timing for maximized reproduction success. PDF is also necessary for the copulation process by enabling the critical coordination of muscles necessary for the successful transfer of sperm cells. PDF is produced in the CNS, particularly in the small and large ventrolateral neurons of the brain projecting to the dorsal protocerebrum, mushroom bodies, and neuroendocrine centers. Its signaling mechanism involves the PDF receptor (PDFR), a class B GPCR [128]. When PDF binds to PDFR, it triggers the increase in cAMP, which activates PKA (protein Kinase A), which also triggers the CREB (cAMP Response Element-Binding protein) pathway to modulate circadian clock genes, ion channel activities and mating-related channels [128]. Depending on the time of the day, the CREB pathway may signal sexual receptivity, discrimination of a mate, or the release of JH or Ecdysis Triggering Hormone (ETH) hormones. PDF sequence and its receptor architecture are strongly conserved in many insect orders and crustaceans, suggesting its vital role in circadian and behavioral integration for the reproduction and perpetuation of species.

### 3.2. Role in Copulation

#### 3.2.1. Adipokinetic Hormone (AKH)

Adipokinetic hormone (AKH) is a neuropeptide critical in the energy mobilization in insects and is dubbed a “hormone of all seasons” [129]. Structurally, it is a small peptide comprising 8–10 amino acids. Functionally similar to vertebrates’ glucagon, AKH mobilizes energy stores during high metabolic demand, like flight, starvation, or reproduction activities such as copulation. Critically, mating requires the mobilization of energy stores, which is triggered by AKH [85]. AKH is produced from special neurons found in the corpora cardiaca, and its secretion is triggered by nutritional status, circadian rhythms, and environmental stress [129]. It is expressed in the fat body and various neurons [130]. AKH functions by binding to its receptor, AKHR, a GPCR that triggers the Gq pathway to mobilize energy substrates such as lipids [131]. The Gq pathway mediates the activation of phospholipase C (PLC), which cleaves PIP2 into IP_3_, which induces Ca^2+^ release, and DAG, which activates PKC. Ca^2+^ and PKC stimulate energy mobilization. Moreover, AKHR triggers cAMP/PKA signaling via the Gs pathway, leading to lipase activation. AKH is conserved across arthropods and bears structural similarity with the gonadotropin-releasing hormone (GnRH) in vertebrates, suggesting a shared ancestry.

#### 3.2.2. Corazonin (Crz)

Corazonin (Crz) is an 11-amino-acid neuropeptide occurring in arthropods [132]. It is a homolog of the mammalian Gonadotropin-Releasing Hormone (GnRH) produced in the CNS, specifically in the neurosecretory cells of the brain and ventral nerve cord [132]. The Crz neurons are expressed in the pars lateralis, lateral protocerebrum, median neurosecretory cells, and abdominal ganglia, projecting into centers of sexual behavior, hormone production, and motor and courtship centers of the ventral nerve cord. Crz is highly conserved, regulating a diversity of physiological roles, including sexual drive. Studies have shown that Crz modulates the duration of copulation, ejaculation, and reward behaviors [132,133]. Crz binds to a G-protein-coupled receptor (CRZR), which is a rhodopsin-like GPCR [134]. CRZR triggers the Gq proteins signaling pathway, which activates phospholipase C (PLC), resulting in intracellular accumulation of Ca^2+^ mobilization, consequently triggering the PKC (Protein Kinase C) pathway, which initiates behavior change expression [134]. Crz interacts with hormones, including JH and ecdysone, to modulate behavior. Crz is highly conserved across insects, with the pQTFQYSRGWTNamide core being maintained across species, suggesting its functional importance.

#### 3.2.3. Short Neuropeptide F (sNPF)

Short neuropeptide F (sNPF) is a versatile neurohormone regulating several insect physiological functions, including feeding, reproduction, development, and circadian rhythms [3,135]. sNPF is generated in the CNS, primarily in the brain and ventral nerve cord, released by specific interneurons and neurosecretory cells, and widely expressed in the olfactory processing centers, mushroom bodies, lateral protocerebrum, and the abdominal ganglia [136,137]. Crucial for its functions, sNPF has a C-terminal motif (X-X-X-X-RFamide) with a highly conserved amidated RF. sNPF occurs in a wide range of species, and its precursor molecule can have anywhere from one to four isoforms [135]. To function, sNPF binds to its G-protein-coupled receptor (sNPFR). Typically, sNPFR activates the Gq signaling pathway, which activates PLC/IP3/Ca^2+^ cascades, leading to downstream behavior decisions. However, sNPFR may also activate the Gi/o protein pathway, which inhibits cAMP production and downregulation of PKA while regulating ion channels and other effectors responsible for behavior change. Moreover, the sNPF signaling pathway involves interactions with other neuropeptides and hormonal pathways, including PDFs, SIFa, ILPs, JH, and ecdysteroids, to regulate various physiological processes [135]. sNPF is key in modulating sex drive, copulatory persistence, and sexual satiety. Male *D.melanogaster* exhibit a shorter mating duration based on sexual satiety, a phenomenon referred to as shorter mating duration (SMD) that is dependent on sNPF signaling [138]. The structural motif RFamide of sNPF is evolutionarily conserved in coleopterans, dipterans, hymenopterans, and hemipterans, pointing to its critical modulatory role.

### 3.3. Role in Post-Mating Behaviors

Neuropeptides play a role in controlling the reproductive physiology and behavior of females after mating, including oviposition and decreased susceptibility to remating. Peptides transferred in the seminal fluid cause post-mating responses (PMR) in many female insects, including a sharp rise in egg-laying and a decrease in mating receptivity [139].

#### 3.3.1. Sex Peptide (SP)

Sex Peptide (SP) is a small 36-amino-acid peptide found in *D. melanogaster*. It is produced in the male accessory glands and transferred from males to females via seminal fluid during copulation to inhibit remating as PMR and to stimulate a switch from mating to feeding and oviposition [85,140,141,142,143]. After mating, SP triggers an array of behavioral, physiological, and hormonal changes and is widely expressed in the female reproductive tract, specific sensory neurons, and central brain circuits. SP exerts its effects through multiple pathways, including its receptor, SPR, and interaction with other pathways. SP may elicit short or long PMR (also called “sperm effect”) in female *D. melanogaster*, depending on the availability of sperm [139]. For sperm retention in storage, SP binds to a sperm cell with its C-terminal end and is gradually released by cleavage at the trypsin cleavage site inside the female reproductive tract, thus initiating or prolonging PMR [139]. SPR (GPCR receptor) activation may trigger the Gi/o signaling pathway that mediates the inhibition of cAMP and downregulation of PKA, leading to downstream changes in gene expression [144]. Inside the female uterus (uterine epithelium), SP may also bind to SPR on the sex peptide sensory neurons (SPSNs) that project into the abdominal ganglion and the brain, leading to the alternation of NPF, octopamine, and ILP signaling, resulting in behavior change, hormonal modulation, and egg maturation. SPR receptor structure and PMR pathway are highly conserved across *D. melanogaster* species, suggesting a strong evolutionary pressure geared towards maintaining its role in reproduction.

#### 3.3.2. Diuretic Hormones (DH44)

Diuretic hormone DH44 is structurally composed of 44 amino acids, is homologous to vertebrate Corticotropin-Releasing Factor (CRF), and is central to modulating body fluid homeostasis, metabolic processes, and sleep–wake cycles (circadian rhythms), including reproduction and mating behaviors [94,145]. It is widely studied in *D. melanogaster*; however, its homologs occur in a range of other insect orders. DH44 modulates oviposition and fluid regulation in reproductive tissues, particularly coordinating with SP and JH to regulate fluid homeostasis during oviposition. The DH 44 neuronal circuit comprises six neurosecretory cells in the pars intercerebralis projecting to the corpus cardiacum, ventral nerve cord, and abdominal ganglia. It is released from median neurosecretory cells (MNCs) in the brain and acts on malpighian tubules, gut, and brain centers to exert its functions [145]. Its mechanistic aspects involve two receptors, DH44-R1 and DH44-R2. Both are GPCRs, but they differ in expression patterns [146]. When DH44 binds to its receptor, it triggers the Gs pathway, which leads to an increase in cAMP, which activates the PKA pathway, consequently stirring downstream effects [145]. In some tissues, DH44 may bind to its receptor, triggering Gq pathway, which leads to Ca^2+^ accumulation that activates the PKC pathway and stirs downstream effects. However, it is not known how DH44 interacts with sex-specific circuits to modulate post-mating behaviors. In female *D. melanogaster*, DH44 influences the ejection and storage of sperm in the uterus [146]. In a study, it was observed that in 1–6 h, female *D. melanogaster* ejected male ejaculates from the uterus, and the action was controlled by a brain-signaling system made up of DH44. It was further observed that whereas DH44 signal enhancement delayed sperm expulsion, DH44 signal suppression in the brain accelerated sperm ejection from the uterus, leading to a significant drop in sperm in the storage organs, thus lowering fecundity [146]. DH 44 and its receptors are conserved across insect orders, including Diptera, Coleoptera, Hemiptera, and Lepidoptera, indicating a strong evolutionary pressure to maintain the tradeoff between reproduction and survival in insects.

#### 3.3.3. Insulin-like Peptides (ILPs)

Insulin-like peptides (ILPs) are essential for insect metabolism, development, reproduction, and aging [147]. They function through a conserved insulin signaling mechanism and influence several physiological processes (oogenesis, ovulation, and sperm storage) necessary for egg production [148,149,150,151]. ILPs are analogous to vertebrate insulin and insulin-like growth factors (IGFs) both structurally and functionally. ILPs are produced in the insulin-producing cells (IPCs) located in the pars intercerebralis and project to mushroom bodies, GnRH-like centers, and circadian centers [152]. ILP binds to its receptor (InR) to activate downstream signaling cascades, including PI3K/Akt (Phosphoinositide 3-Kinase/Protein Kinase B), MAPK, and TOR pathways that are responsible for promoting different responses [152]. ILP signaling also interacts with endocrine factors, including JH, AKH, and ecdysteroids, to exert its function [152]. To ascertain ILPs’ functions, a study by Chen and Dou [153] revealed that ILPs interacting with ovary ecdysteroidogenic hormone (OEH) promoted egg development and food storage in *Aedes aegypti* mosquitoes. Similarly, injecting bovine insulin into female adults of green lacewing (*Chrysopa septempunctata*) resulted in increased protease activity, ovarian development, Vg protein abundance, and elevated reproductive performance [147]. ILPs, together with tyrosine kinase receptor (InR), are highly conserved across insects, crustaceans, nematodes, and vertebrates, with conserved downstream signaling cascades pointing to evolutionary pressure to maintain their role for survival.

#### 3.3.4. Ion Transport Peptide (ITP)

Ion transport peptide (ITP) and its variant ITP-like (ITPL) have a variety of functions in the physiology of insects and crustaceans, including osmotic balance, feeding, and reproductive physiology [154,155]. In a study on mosquitoes (*A. aegypti*), the knockdown of ITP and ITPL led to a decrease in blood feeding, egg laying, and egg viability, indicating their significance in reproductive physiology and behavior [156]. Similarly, a knockdown of ITP in the female adults of the red flour beetle (*T. castaneum*) was shown to cause a decrease in egg production [157]. ITP is secreted in the median neurosecretory cells in the brain and projects to neurohemal organs, e.g., corpora cardiaca, digestive tract, Malpighian tubules, and the reproductive tissues. To exert its function, ITP binds to corticotropin-releasing factor (CRF)-like receptor, a Class B1 family of GPCRs. For instance, in locusts, ITP binds to ITPR to trigger the increase in cAMP/PKA, consequently activating ion transporters downstream, targeting V-ATPase for ion secretion in the Malpighian tubules and Na^+^/K^+^-ATPase to maintain electrochemical gradients [158]. During stress-induced diuresis, ITP also synergizes with DH44 to stabilize osmotic balance, and during favorable conditions, it also synergizes with AKH to streamline reproductive behavior [155]. ITP is widely and functionally conserved in insects for osmoregulation and reproductive support.

#### 3.3.5. Sulfakinin (SK)

Sulfakinin (SK) is primarily found in insects but is analogous to cholecystokinin (CCK) in vertebrates, both in structure and function [159]. SKs are highly conserved with a characteristic C-terminal tyrosine residue (Y(SO_3_H)) and FMRFamide heptapeptide core [160]. It exists in two primary forms, short sulfakinin (sSK) and long sulfakinin (lSK), which differ in their peptide lengths. The key function of SK is to regulate feeding and digestion in insects [160]. Upon mating, insects switch into foraging mode to find food to enhance their survival. The switching from mating to foraging is mediated by SK [123,161]. In a study, the Oriental fruit flies (*B. dorsalis*) were shown to suppress their response to opposite-sex pheromones while increasing their sensitivity to food odors. Even though the flies (*B. dorsalis*) were starved, their antennal responses changed to mating mode when the gene encoding SK receptor 1 (SkR1) was knocked out, suggesting that SkR1 suppressed mating but promoted foraging [161]. Similarly, SK was found to suppress mating behavior in *D. melanogaster* through its receptor CCKLR-17D3 [123,162]. SK is produced in the CNS, particularly the brain, and expressed in the various parts of the CNS and peripheral tissues, including the midgut and fat body. In *D. melanogaster*, SK is expressed in a small number of SK-expressing neurons (SKNs) in the subesophageal zone (SEZ) and pars intercerebralis [159]. The modulation function of SK is mediated through the G-protein-coupled receptor signaling system. SK binds to the GCPRs, SKR1 and SKR2, the two types that occur in *D. melanogaster*. Binding activates the Gq signaling pathway downstream, which triggers PLC/IP_3_/Ca^2+^ second messenger pathways, consequently affecting behavioral expression [160]. In some other contexts, the Gq signaling pathway triggers the cAMP/PKA second messenger pathway [161]. SK is widely conserved in insects, with its presence in coleopterans, hemipterans, dipterans, orthopterans, and hymenopterans, striking a parallel function with its homology CCK in vertebrates [160]. Its role of trading mating to feeding points to an evolution strategy that prioritizes survival over reproduction during unfavorable conditions.

## 4. Discussion of Findings

**Figure 3:**
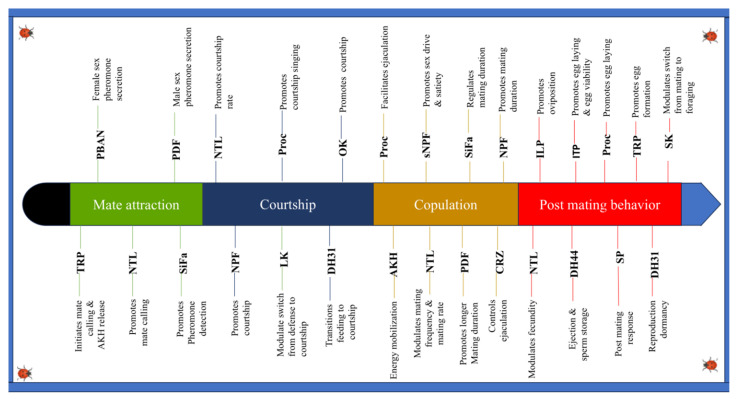
Neuropeptides play a crucial role in driving insect mating behaviors. Without TRPs, the distinctive, loud chorus of cicadas—heralding the start of summer—might be absent from nature. Similarly, without AKH, which mobilizes energy reserves for mating, many insect species could have faced extinction by now. Created in BioRender. guo, j. (2025) https://BioRender.com/hggt62j (accessed on 28 April 2025).

Insect mating is a continuum of behaviors that range from mate attraction and courtship rituals to copulation and post-mating actions. The complex behaviors vary amongst species, propelled by an intricate underlying neuropeptidergic mechanism. Insects possess a variety of neuropeptides that drive the mating process. Whether it is the release of sex pheromones by moths, the stunning courtship displays of dragonflies, the beautiful melodies of cicadas and crickets, the amazing indirect copulation of springtails, or the prolonged copulatory contact of damselflies, these diverse mating behaviors are all modulated by the neuropeptides. In this review, we highlight 18 neuropeptides that we found to influence behavior in the different stages of insect mating, as elaborated in Figure 3.

As demonstrated (Figure 3), some neuropeptides are multifunctional, while others are specific. For instance, NTL influences mate attraction and courtship in the fall armyworm (*S. frugiperda*) and increases mating appetite in the oriental fruit fly (*B. dorsalis*) and egg-laying in the red flour beetle (*T. castaneum).* Hence, it is the most versatile neuropeptide [49,163], unlike PBAN, which regulates the production of sex pheromones in moths only [70].

**Figure 4:**
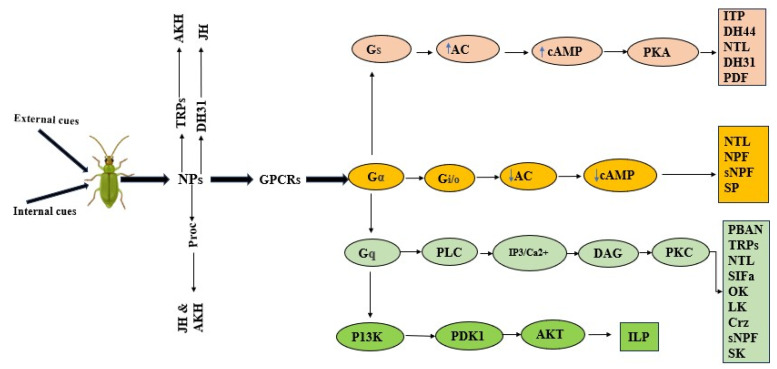
The illustration shows the GPCR signaling pathway exploited by neuropeptides in insects to transmit signals regulating various physiological processes, including the mating behaviors. Neuropeptides are triggered by internal or external cues, and they act either by binding to the GPCRs, triggering heterotrimeric G protein signaling pathways and downstream effectors that cause behavioral expression or via the endocrine system to trigger hormonal responses for regulating specific behavior. Created in BioRender. guo, j. (2025) https://BioRender.com/jnf64vj (accessed on 28 April 2025).

Most neuropeptides are produced in the CNS, primarily the brain and the ventral nerve cord. However, some neuropeptides, for instance, TRP, can be released as a circulating hormone from midgut endocrine cells of locusts and cockroaches during fight or flight stress [82]. SP is also produced in the male accessory glands and delivered into the female reproductive system, while AKH is partly produced in the corpus cardiacum (CC). Neuropeptides execute their regulatory functions through the G protein-coupled receptor (GPCR) signaling pathway, which comprises receptors (GPCRs), heterotrimeric G proteins, downstream effectors, and messengers, as shown in Figure 4.

Heterotrimeric G Proteins are the Guanine nucleotide-binding proteins Gα, Gβ, and Gγ subunits. Insects, however, largely utilize Gα in the signaling process comprising Gs, Gi/o, and Gq subunits, as shown in the illustration (Figure 4). The subunits activate downstream effectors, including Adenylate Cyclase (AC) and Phospholipase C (PLC). However, unlike other neuropeptides using the Gq pathway, ILPs utilize P13K as the downstream effector. The messengers in the Gq pathway comprise cAMP, which activates Protein Kinase A (PKA), and Ca^2+^ and DAG, which activate Protein Kinase C (PKC). As shown, the majority of the neuropeptides reviewed in our work exploit the Gq signaling pathway; however, NTL is capable of exploiting any of the three pathways, possibly the reason behind the ability to mediate more than one activity in the insect mating process. Neuropeptides activate signaling pathways that cause changes in neuronal activity, while others regulate the production of hormones responsible for a certain physiological function [135]. TRPs induce the release of AKH in locusts, while PBAN induces the production of sex pheromones in moths [70,82]. In certain situations, neuropeptides work together to modulate a certain function. For instance, SIFa and sNPF neurons work together in a signaling mechanism that modulates the mating duration to modify behaviors like SMD and LMD.

To modulate some mating behaviors, neuropeptides work with other systems, including the endocrine system [164]. In female *D. melanogaster*, DH31 suppresses the production of JH, triggering reproductive dormancy when conditions are not favorable [96]. Reproductive dormancy is critical in prioritizing feeding over reproduction for enhanced survival. In addition, ILPs, in conjunction with JH and ecdysteroids, influence physiological processes like oogenesis, ovulation, and sperm storage that are necessary for egg production [150]. Based on such interactions, the neuropeptide signaling systems on physiological processes can be interrupted to form suitable targets for sustainable pest management tools.

In response to the varied and sometimes fluctuating ecological environments, insects evolve to maximize reproductive and survival success. Pressures, including mate competition, predation, harsh environments, limited resources, and reproductive methods, all influence these evolutionary adaptations. Neuropeptides play a critical role in maintaining critical functions necessary for survival and the perpetuation of species. The neuropeptides reviewed in our work consistently show conserved motifs in their structures responsible for certain behaviors. For instance, the C-terminal motif of FXPRLamide in PBAN is structurally conserved in the lepidopteran females to maintain its mate attraction function across the species [72].

Although their processes, effects, and evolutionary adaptations differ greatly, insect neuropeptides play similar roles as other neuropeptides, including their homologs in vertebrates. For instance, in *D. melanogaster*, SP is essential in modulating post-mating behaviors by discouraging females from remating and promoting transition into oviposition [139]. In long-term relationships, neuropeptides such as vasopressin and oxytocin control sexual receptivity and pair bonding in higher animals [165].

## 5. Conclusions and Future Perspectives

As incredibly varied creatures, insects display a range of mating behaviors meticulously regulated by neuropeptides. The 18 neuropeptides synthesized in this review play a critical role in the mating process of various insects. Their interaction with hormones and neurotransmitters ensures successful reproduction and perpetuation of the species. The mechanisms, routes, and evolutionary adaptations underlying the regulation of mating behavior in many insects remain largely unknown despite the notable advancements in identifying important neuropeptides and their roles. From this review, we note that most of what is known about these important neuropeptides is based largely on research on fruit flies (*D. melanogaster*). There is a wide range of mating behaviors in many other species whose neuropeptidergic regulatory mechanisms remain unknown. There is a need to replicate research in other species, especially on insects emerging as invasive and destructive globally.

We also note that the research conducted on the functions of neuropeptides, as elucidated in this review, is largely based on RNAi knockdown in conjunction with behavioral assays. Neuronal circuit analysis based on the latest and more robust tools could provide a deeper understanding of the currently unknown mechanistic aspects. Electron microscopy (EM) is potentially a powerful tool in the construction of connectomes that can precisely unravel neuronal pathways, specific targets, and the synaptic architectures involved in mediating mating behavior. For instance, using EM connectomes, expressing neurons of OK can be traced, including the downstream targets and feedback loops, to provide an understanding of the signaling mechanism that is currently not well understood. After mating in *D. melanogaster*, male-derived SP triggers an array of changes in the female to prevent remating [139]. Synaptic changes after mating that block receptivity can be established based on EM connectomes. EM has previously been applied in *D. Melanogaster* to identify the neuronal circuit in the visual system that computes motion signals [166]. Functional imaging using genetically encoded calcium indicators (e.g., GCaMP) could also be deployed to study the neuronal circuits of neuropeptides in real time. For instance, the blocked remating phenomenon in *D. melanogaster* females could be studied in real time, such as how SP silences the pickpocket ion (ppk+) sensory neurons in females to cut the responses to courtship songs. Single-cell RNA-sequencing (scRNA-seq) could be deployed to compare species and provide evolutionary insights. Neuropeptides, for instance, PBAN, are highly conserved in the lepidopteran species, performing a similar function in mate attraction. scRNA-seq could be deployed in studies to identify receptor expression differences that prevent cross-species courtship and mating. With its chemical specificity, sensitivity, and quantitative precision, mass spectrometry (MS) is a robust tool for researching neuropeptide dynamics in insect mating behavior. Firstly, MS could be critical in characterizing new neuropeptides that are yet to be known to mediate insect mating behavior. This is specifically important because the current research on neuropeptides has been conducted on the model insect *D. melanogaster*, with many insects not yet touched. MS could also be critical in the study of how neuropeptide quantity levels in the insect hemolymph relate to mating behaviors. For instance, MS could be deployed to study how the quantity of SP in the female *D. menologaster* affects receptivity to remating versus time after the completion of initial mating.

Neuropeptides are potential targets for novel insect control techniques because of their significance in reproduction and survival [4]. Targeted manipulation of the signaling system of the neuropeptides will disrupt mating behaviors and other physiological processes that sustain the reproduction and survival of some economically disruptive pests. Tools applied to disrupt the signaling system in the mating process would be applied in crop production systems to reduce the population pressure of destructive pests, thus offering ecologically friendly substitutes to the conventional use of chemical pesticides. Our findings in this review not only advance the understanding of neuropeptide-mediated mating regulation but also highlight their potential as eco-friendly pest control targets.
